# Shear Bond Strength of Additively and Subtractively Manufactured CAD/CAM Restorative Materials After Different Surface Treatments and Adhesive Strategies: An In Vitro Study

**DOI:** 10.3390/polym18020296

**Published:** 2026-01-22

**Authors:** Sevim Atilan Yavuz, Ayse Tugba Erturk-Avunduk, Omer Sagsoz, Ebru Delikan, Ozcan Karatas

**Affiliations:** 1 Department of Restorative Dentistry, Faculty of Dentistry, Mersin University, Mersin 33190, Türkiye; aysetugba@mersin.edu.tr; 2 Department of Restorative Dentistry, Faculty of Dentistry, Atatürk University, Erzurum 25240, Türkiye; omer.sagsoz@atauni.edu.tr; 3 Department of Pediatric Dentistry, Faculty of Dentistry, Nuh Naci Yazgan University, Kayseri 38100, Türkiye; edelikan@nny.edu.tr; 4 Department of Restorative Dentistry, Faculty of Dentistry, Nuh Naci Yazgan University, Kayseri 38100, Türkiye; okaratas@nny.edu.tr

**Keywords:** additively manufactured, adhesive systems, shear bond strength, subtractively manufactured, surface treatment

## Abstract

This study aims to evaluate the effects of different surface treatments and adhesive systems on the shear bond strength (SBS) of additively manufactured (AM) and subtractively manufactured (SM) restorative materials. A total 675 rectangular specimens of three AM (Saremco Crowntec/SC, VarseoSmile CrownPlus/VC, and VarseoSmile TriniQ/VT) and two SM (Vita Enamic/VE and Cerasmart/CS) restorative materials were fabricated. Each material was randomly divided into three groups regarding surface treatments: control/C, sandblasting/S, and etching/E. Following surface treatments, each AM and SM restorative material was then divided into three subgroups (15 specimens/subgroup) on the basis of adhesive systems (etch-and-rinse, self-etch, and universal). All specimens were thermocycled at 10,000 cycles, 5–55 °C, 30 s dwell time, and tested under SBS until failure, and failure types were examined under a stereomicroscope. Representative specimens were examined by SEM to evaluate fracture morphology. Statistical analysis was set at *p* < 0.05. There were significant differences in bond strength according to the material, surface treatment, adhesives, and their interactions (*p* < 0.05). The highest SBS value was obtained with SC × sandblasting × etch-and-rinse (16.45 ± 0.93 MPa), while the lowest value was found in the CS × control × universal interaction (4.68 ± 1.1 MPa). Outcomes varied according to the materials, surface treatment, and adhesive strategy. Clinically, these findings indicate that SM materials may require various surface treatment to achieve reliable adhesion, whereas AM materials provide more similar bond strength performance with no surface treatment.

## 1. Introduction

Computer-aided design/computer-aided manufacturing (CAD/CAM) technologies have transformed restorative dentistry by improving accuracy, reproducibility, and material efficiency. Two major CAD/CAM fabrication routes dominate dental practice: additively manufactured (AM) and subtractively manufactured (SM) [1]. Subtractive manufacturing removes material from prefabricated blocks, resulting in restorations with high precision and predictable mechanical performance [1]. In contrast, AM builds restorations layer-by-layer and enables cost-effective workflows with minimal material waste [2]. AM materials are predominantly resin-based systems that undergo photopolymerization during a fabrication process [3]. Unlike SM materials, which are industrially polymerized under controlled pressure and temperature conditions, AM materials may exhibit a lower degree of conversion and increased anisotropy depending on printing parameters such as layer thickness, build orientation, and post-curing protocols [4]. These factors can result in variations in mechanical behavior, surface morphology, and interlayer bonding, potentially affecting the bonding performance of AM restorations [5]. In addition, the presence of residual monomers and oxygen-inhibited layers on the surface of AM materials may alter their interaction with adhesive systems, thereby influencing micromechanical retention and chemical adhesion [6].

Although AM and SM restorative materials offer several advantages, failures such as chipping or bulk fracture may still occur as a result of inadequate bonding to the tooth structure, occlusal stress, intrinsic porosities related to the manufacturing technique, or parafunctional habits [7]. Completely replacing a defective restoration is often not the most conservative or practical solution, as it increases treatment costs and prolongs chairside time [8]. Since the repair of indirect restorations is frequently preferred in clinical practice, surface treatment and adhesive system selection, which are among the repair stages, are of critical importance [9].

A clinically reliable SBS between restorative materials and resin composites necessitates the establishment of both optimal micromechanical retention and stable chemical adhesion at the material interface [10]. Surface treatment is therefore a critical step to enhance surface energy, wettability, and micro-retentive features [11]. Among commonly used protocols, hydrofluoric acid (HF) modifies the ceramic-rich phase of hybrid materials by selectively dissolving glassy components, thereby increasing porosity and facilitating resin infiltration [12]. Although, sandblasting with aluminium oxide particules (Al_2_O_3_) creates micro-undercuts through controlled abrasive impact, improving mechanical interlocking and promoting predictable bonding in both SM and AM materials [13]. Many studies have shown that surface treatment strongly influences SBS values in AM and SM restorative materials [11,13].

In addition to surface treatment, adhesive systems play a critical role in determining the quality and durability of SBS [14]. Conventional etch-and-rinse adhesive systems primarily rely on micromechanical interlocking achieved by phosphoric acid etching followed by resin infiltration, providing effective bonding but with technique sensitivity and increased clinical steps [15]. In contrast, self-etch adhesive systems incorporate acidic functional monomers that simultaneously condition and prime the substrate, reducing postoperative sensitivity and simplifying clinical application; however, their relatively mild etching potential may limit micromechanical retention, particularly on highly polymerized CAD/CAM materials [15,16]. The development of universal adhesives has expanded clinical versatility by allowing for their use in etch-and-rinse, self-etch, or selective-etch modes [15]. Notably, universal adhesives containing 10-methacryloyloxydecyl dihydrogen phosphate (10-MDP), enable additional chemical bonding through stable interactions with metal oxides, zirconia, resin composites, and hybrid ceramics, potentially enhancing bond durability [17]. Nevertheless, the literature remains limited regarding the comparative effects of different adhesive strategies on the SBS of AM and SM restorative materials subjected to various surface treatments, underscoring the need for further investigation to guide optimal adhesive system selection.

Although glass–ceramics are commonly used for indirect restorations, the present study focuses on resin-matrix ceramics and additively manufactured resins. Therefore, subtractively manufactured hybrid materials (Vita Enamic and Cerasmart) were selected as reference materials to enable a comparable evaluation of polymer-based microstructures [18]. Moreover, many studies have evaluated bonding protocols for only SM materials; no previous investigation has simultaneously compared multiple AM and SM restorative materials combined with three surface treatments and three adhesive strategies within a unified experimental design [19,20]. Comprehensive data on the bonding behavior of AM materials used as permanent restoration resins, such as Saremco Crowntec, VarseoSmile TriniQ, and VarseoSmile CrownPlus, is still limited. Therefore, the present study aimed to evaluate the SBS of five restorative materials—three AM and two SM—after three surface treatments and three adhesive systems.

The null hypotheses of the current study were tested:

(1) The restorative materials have no significant effect on SBS;
(2) The surface treatment protocols have no significant effect on SBS;
(3) The adhesive systems have no significant effect on SBS.

## 2. Materials and Methods

### 2.1. Restorative Materials Used in the Study

In the present study, three AM (Saremco Crowntec/SC, VarseoSmile CrownPlus/VC, and VarseoSmile TriniQ/VT) and two SM (Vita Enamic/VE, and Cerasmart/CS) CAD/CAM restorative materials were evaluated. The materials used in this study, manufacturer, composition, and LOT number are listed in Table 1.

### 2.2. Specimen Preparation

**Figure 1:**
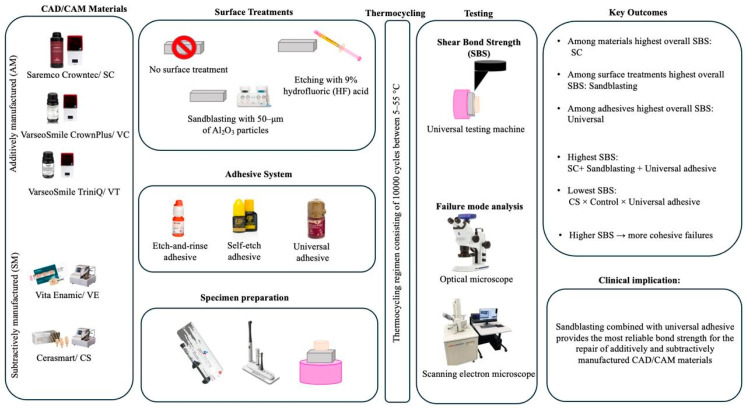
The graphical abstract of the study.

An a priori power analysis was performed using G*Power software (Version 3.1.9.4. Heinrich Heine University, Düsseldorf, Germany) based on a fixed-effects one-way ANOVA model. The effect size (f = 0.1373) was calculated using mean and standard deviation values reported by Mao et al. [19], who investigated surface treatment effects on the bond strength of additively and subtractively manufactured hybrid materials. Using α = 0.05 and power = 0.90, the required total sample size was estimated as 675 specimens in this study. A summary diagram of the study is given in Figure 1 as a graphical abstract.

The AM specimens were designed with a dimension of 7 × 6 × 2 mm in an Exocad CAD software (Elefsina v3.2, Exocad GmbH, Darmstadt, Germany; Dental CAD Rijeka). Afterwards, the design files were exported to the standard tessellation language (STL) format. This STL file was transported into a nesting software (Composer v1.3.3; Asiga, Sydney, NSW, Australia) and positioned on its flat surface. The specimens were fabricated by digital light processing (DLP) using a 3-dimensional (3D) printer (Asiga Pro 2–75, Alexandria, Australia). All AM specimens were printed at an angle of 90 degrees on the build platform and with a layer thickness of 50 μm without supports [19]. According to the manufacturer’s instructions, after the printing process, SC specimens were cleaned with an alcohol-soaked (96%) cloth until all resin residues were completely removed, while VC and VT specimens were ultrasonically cleaned in ethanol for 5 min (3 min of precleaning in reusable ethanol and an additional 2 min in fresh ethanol). Specimens were then air-dried and light-polymerized either with 4000 (SC, 2 × 2000) or 3000 (VC and VT, 2 × 1500) light exposures (Otoflash G171; NK Optik, Baierbrunn, Germany) under a nitrogen oxide gas atmosphere.

For SM specimens, 7 mm × 6 mm × 2 mm plates were prepared from SM blocks (n = 135 per SM restorative material) by using a low-speed diamond saw (IsoMet 1000; Buehler Ltd., Lake Bluff, IL, USA) under water cooling. A digital caliper (N48AA, Maplin Electronics, Rotherham, UK) was used to ensure that the final thickness of the specimens was 2 ± 0.1 mm. To standardize the bonding surfaces, all specimens were finished with 600-, 800-, and 1200-grit silicon carbide abrasive paper for 20 s under continuous water irrigation.

The finishing procedure was applied to only one surface of each specimen. To ensure standardization and to avoid treating the wrong surface, the opposite (non-treated and non-debonded) side of each specimen was immediately marked after polishing. The specimens were embedded in plastic molds using cold-cure acrylic resin (Imicryl™, Konya, Türkiye), leaving the surface exposed.

### 2.3. Surface Treatment

Based on the surface treatments, the specimens were divided into three groups:

Group Control/C: No surface treatment was applied.

Group Etching/E: The specimen surfaces were etched with 9% HF (Ultradent, South Jordan, UT, USA) for 60 s, rinsed with water for 20 s, and then dried using air spray [19,21].

Group Sandblasting/S: Specimen surfaces were sandblasted with 50 μm of aluminum oxide (Al_2_O_3_) particles at 2 bar pressure for 10 s at a 10 mm distance with a circular motion. A permanent marker was used to paint the targeted surface. Sandblasting was performed using the AquaCare Twin Airflow and Intraoral Sandblasting Device (Velopex International, London, UK).

### 2.4. Adhesive Procedure and Shear Bond Strength (SBS)

All adhesive procedures (etch-and-rinse, self-etch, and universal) that were performed following the manufacturer’s instructions are provided in Table 1. Fifteen specimens were used for each subgroup. The single-shade resin composite (ZenChroma, FGM Dental, Joinville, SC, Brazil) was placed onto the prepared surfaces of the AM and SM restorative material specimens using a plastic tube mold (approximately 2 mm in height). The composite was incrementally condensed within the mold to ensure proper adaptation to the substrate, and subsequently light-cured for 20 s using a light-emitting-diode curing unit (LED, SmartLite Pro, Dentsply DeTrey GmbH, Constance, Germany) with an irradiance of 1200 mW/cm^2^. All bonded specimens for each group were stored in distilled water at 37 ± 1 °C for 24 h. Subsequently, a thermocycling regimen, consisting of 10,000 cycles between 5 and 55 °C, with a 30 s dwell time at each bath and 15 s transfer time to simulate intraoral aging, was conducted [22].

The specimens were tested for bond strength on a universal testing machine (Inston 3344, Instron Corp., Wilmington, DE, USA) with a crosshead speed of 1 mm/min. The blade tip was subjected to a shear load at the interface between the restorative material and the resin composite, and the maximum load was recorded immediately before fracture occurred. Bond strength was calculated using the formula below:

Bond Strength (MPa) = Fracture Load (N)/Bonded Area (mm^2^) (where N/mm^2^ = MPa).

All tests were performed by a single operator to minimize variability.

### 2.5. Stereomicroscope and Scanning Electron Microscope (SEM) Analysis

To identify the type of failure, the debonded surfaces of both the AM and SM restorative materials and the resin composite cylinders were examined under a stereomicroscope (Zeıss Opmi pıco, Carl Zeıss Meditec AG, Jena, Germany) at ×16 magnification. Based on the proportion of each substrate visible on the fractured surfaces (adhesive, restorative material, or resin composite), failure types were categorized as follows: (1) adhesive failure; (2) cohesive failure within the restorative materials; (3) cohesive failure within the resin composite; or (4) mixed. 

The debonded specimens were mounted on aluminum stubs, sputter-coated, and examined using a scanning electron microscope (SEM, JSM-5600LV, JEOL, Tokyo, Japan) at ×40 magnification to observe the fractured surface topography. 

### 2.6. Statistical Analysis

The data were analyzed using R software (version 4.4.1) and IBM Statistical Package for the Social Sciences version 23 (SPSS, IBM Corp., Armonk, NY, USA). The normality of the data distribution was assessed using the Shapiro–Wilk test. For comparisons based on material, surface treatment, and adhesive factors, a robust ANOVA was performed using the Walrus package for variables that did not meet normality assumptions, followed by Holm-adjusted robust *t*-tests for multiple comparisons. Associations between categorical variables were examined using Monte Carlo/corrected Fisher’s Exact test, and pairwise comparisons were conducted with a Bonferroni post-hoc test. Quantitative data are presented as mean ± standard error, whereas categorical data are expressed as frequencies and percentages. The significance level was set at *p* < 0.05.

## 3. Results

### 3.1. Shear Bond Strength (SBS)

**Figure 2:**
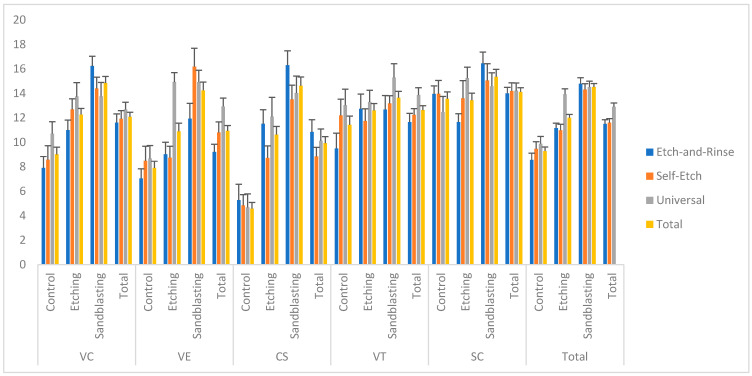
Mean and standard error plot of SBS values based on material, surface treatment, and adhesive factors; Abbreviations: SC: Saremco Crowntec; VC: VarseoSmile CrownPlus; VT; VarseoSmile TriniQ; VE: Vita Enamic; CS: Cerasmart.

Figure 2 and Supplemental Table S1 summarize the mean SBS values obtained for all materials (SC, VC, VT, VE, CS), surface treatments (Control, Etching, Sandblasting), and adhesive systems (etch-and-rinse, self-etch, universal). The robust ANOVA indicated significant main effects of the material (*p* < 0.001), surface treatment (*p* < 0.001), and adhesive system (*p* = 0.002) on SBS. Additionally, all interaction terms—material × surface treatment (*p* = 0.001), material × adhesive (*p* = 0.007), surface treatment × adhesive (*p* = 0.004), and the three-way interaction (*p* = 0.013)—were statistically significant. Among materials, the highest overall SBS mean was observed in SC (14.12 ± 0.35 MPa). Sandblasting produced the highest SBS (14.53 ± 0.27 MPa), followed by etching (12.01 ± 0.27 MPa) and control (9.28 ± 0.34 MPa). Regarding adhesive systems, universal resulted in the statistically significantly highest overall SBS (12.9 ± 0.32 MPa), whereas self-etch (11.6 ± 0.33 MPa) and etch-and-rinse (11.5 ± 0.34 MPa) yielded lower but comparable values. The universal adhesive generally achieved higher bond strengths across multiple material × surface combinations. SC × Sandblasting × etch-and-rinse exhibited the highest SBS value (16.45 ± 0.93 MPa), whereas CS × control × universal displayed one of the lowest (4.68 ± 1.1 MPa).

### 3.2. Failure Type

**Figure 3:**
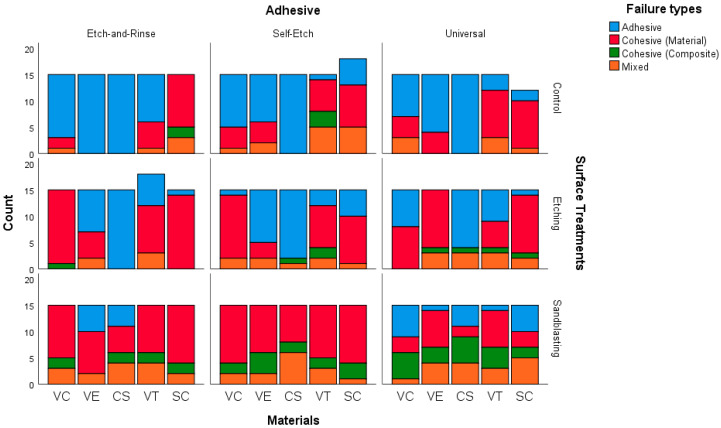
Bar chart investigating the association between adhesive and failure types within material surface treatments. Abbreviations: SC: Saremco Crowntec; VC: VarseoSmile CrownPlus; VT; VarseoSmile TriniQ; VE: Vita Enamic; CS: Cerasmart.

Figure 3 and Supplemental Table S2 present the distribution of failure types (adhesive, cohesive–material, cohesive–composite, and mixed) according to material (SC, VC, VT, VE, CS), surface treatment (control, etching, sandblasting), and adhesive system (etch-and-rinse, self-etch, universal).

In SC, the failure type distribution did not differ significantly under control (*p* = 0.095) or etching (*p* = 0.117), although adhesive failures were generally more frequent under all three adhesive systems. Under sandblasting, a significant difference was detected (*p* = 0.003), with cohesive–material failures predominating across etch-and-rinse, self-etch, and universal. Cohesive–composite failures occurred at lower frequencies regardless of the adhesive system. An analysis of the relationship between adhesive systems and failure types revealed no statistically significant association (*p* = 0.069).

In VC, etching (*p* = 0.001) and sandblasting (*p* = 0.002) demonstrated significant differences. In surface treatment of etching, cohesive–material failures were most dominant in universal adhesive systems (73.3%). Adhesive failures occurred in lower proportions in etching and sandblasting conditions, especially under self-etch and universal adhesives. An analysis of the relationship between adhesive systems and failure types revealed no statistically significant association (*p* = 0.136).

In VT, adhesive failures were significantly more frequent in the Control subgroup (*p* = 0.010), particularly following the etch-and-rinse protocol. For surface treatments of etching (*p* = 0.676) and sandblasting (*p* = 0.847), the distributions were not statistically significant, although cohesive–material failures were relatively frequent, particularly under etch-and-rinse and self-etch adhesives.

In VE, the control subgroup showed statistically significant variation (*p* = 0.037), with a high proportion of adhesive failures in etch-and-rinse. Etching also demonstrated significant differences (*p* = 0.001), dominated by cohesive–material failures (self-etch and universal: 73.3%). Sandblasting was not significant (*p* = 0.069), although cohesive–material failures remained the most common failure mode in all adhesives.

In CS, the control subgroup showed uniform adhesive failure distribution across adhesive systems (all 100%). Surface treatments of etching (*p* = 0.211) and sandblasting (*p* = 0.142) also did not show significant variation. Adhesive failure remained the predominant mode across all adhesives and surface treatments, whereas cohesive–material, cohesive–composite, and mixed failures appeared at much lower frequencies.

In general, regardless of the material, control (*p* = 0.089) and etching (*p* = 0.383) subgroups did not demonstrate significant differences among adhesive systems. Adhesive failures were consistently more common in the control subgroup with the etch-and-rinse, self-etch, and universal adhesive systems, while cohesive-material failures were more common in the etch subgroup. In contrast, sandblasting presented a highly significant difference (*p* < 0.001), characterized by a considerable increase in cohesive–material failures across all adhesive systems.

### 3.3. Assessment of Stereomicroscope and Scanning Electron Microscope (SEM) Analysis

**Figure 4:**
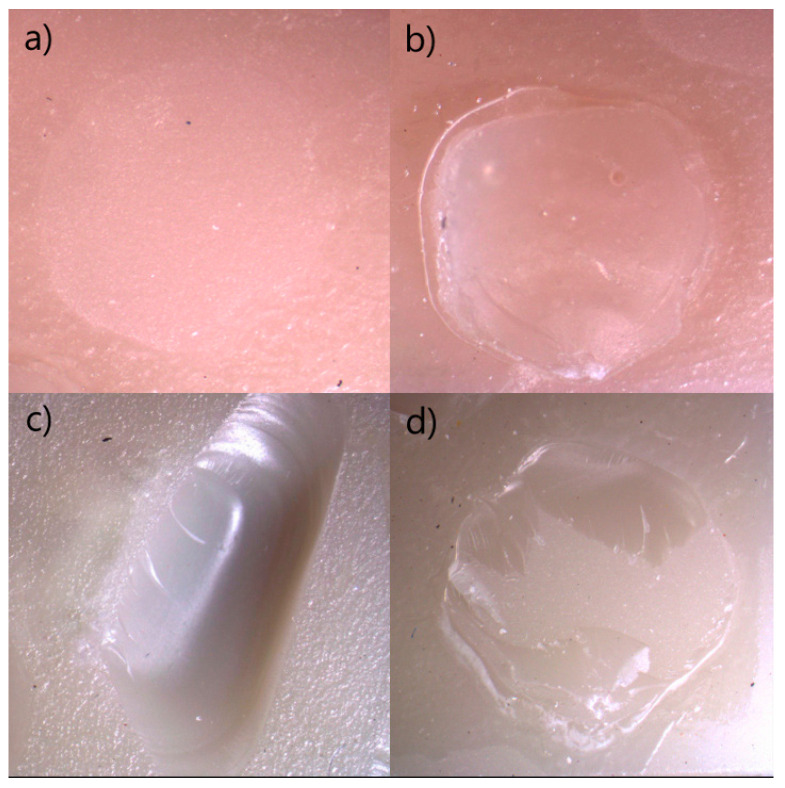
Failure type of debonded surface’s stereomicroscope images. All images were taken at ×16 magnification. (**a**) Adhesive failure type in Group CP × sandblasting × universal adhesive. (**b**) Cohesive composite failure type in Group CP × sandblasting × universal adhesive. (**c**) Cohesive material failure type in Group CT × control × self-etch adhesive. (**d**) Mixed-failure type in Group CT × sandblasting × etch-and-rinse adhesive. Abbreviations: CP: VarseoSmile CrownPlus.

**Figure 5:**
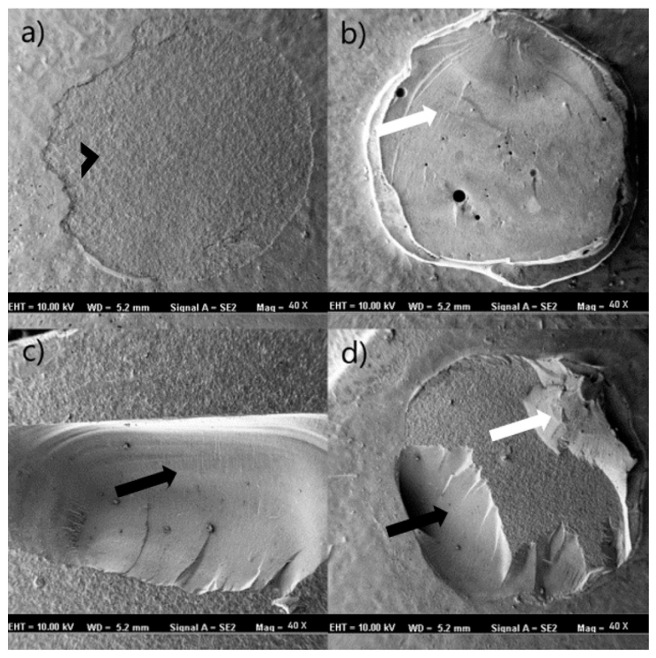
Failure type of debonded surface’s scanning electron microscope (SEM) images. All images were taken at ×40 magnification. White arrows represent resin composite, black arrows represent restorative material, and black arrowhead represents adhesive layer. (**a**) Adhesive failure type in Group CP × sandblasting × universal adhesive. (**b**) Cohesive composite failure type in Group CP × sandblasting × universal adhesive. (**c**) Cohesive material failure type in Group CT × control × self-etch adhesive. (**d**) Mixed-failure type in Group CT × sandblasting × etch-and-rinse adhesive. Abbreviations: CP: VarseoSmile CrownPlus.

Figure 4 presents representative stereomicroscopic images of adhesive, cohesive–material, cohesive–composite, and mixed failure modes observed across different materials, surface treatments, and adhesive systems, while Figure 5 illustrates the corresponding SEM images of the debonded surfaces. The SEM images demonstrated representative adhesive and cohesive failure patterns in the control, etching, and sandblasting subgroups of the evaluated restorative materials. In cohesive failure images, disruption of the structural integrity of either the restorative material or the resin composite was evident. In contrast, adhesive failure images showed an intact restorative surface, with failure primarily localized at the adhesive interface. Mixed failure images exhibited simultaneous features of both adhesive and cohesive failures.

## 4. Discussion

The present in vitro study investigated the shear bond strength (SBS) performance of five restorative materials (SC, VC, VT, VE, and CS) subjected to different surface treatment and adhesive protocols. SBS values differed significantly among the restorative materials. SC exhibited the highest overall bond strength, followed by VT and VC, whereas VE and CS demonstrated comparatively lower values. Therefore, the first null hypothesis of the study, that restorative materials have no significant effect on SBS, was rejected.

SC, VC, and VT, as AM resin-based restorative materials, contain approximately 30–50 wt% inorganic fillers dispersed within a predominantly organic polymer matrix, which is formed through layer-by-layer photopolymerization [21]. This manufacturing approach results in a comparatively lower cross-link density and greater resin phase exposure than that observed in subtractively manufactured materials, potentially facilitating more effective surface modification and enhanced interaction with adhesive monomers [21]. In addition, the resin matrices of AM materials may retain a higher proportion of unreacted functional groups, which could further promote chemical bonding with adhesive systems beyond purely micromechanical retention. Their relatively lower elastic modulus may also allow more favorable stress distribution at the adhesive interface under shear loading, thereby contributing to the higher SBS values observed in these materials [23]. In contrast, VE and CS, as SM hybrid restorative materials, possess highly compact and densely cross-linked microstructures resulting from industrial polymerization and milling processes. VE is a polymer-infiltrated ceramic network (PICN) composed of approximately 86 wt% feldspathic ceramic, while CS contains around 71 wt% barium and silicate glass fillers embedded within a rigid resin framework [21,24]. Although the high inorganic filler content of these materials enhances mechanical durability, hardness, and wear resistance, their dense hybrid architectures may restrict the formation of retentive micro-irregularities and limit effective adhesive penetration [7]. In VE, the coexistence of ceramic and polymer phases may result in heterogeneous surface chemistry, potentially reducing the efficiency of chemical bonding. Furthermore, the higher elastic modulus reported for VE and CS may promote stress concentration at the adhesive interface, which, together with limited surface energy modification and reduced functional monomer infiltration, may explain their comparatively lower SBS values [25]. These findings indicate that the differences in bond strength among the tested materials are not solely governed by surface roughness or micromechanical retention but are strongly influenced by material-specific resin chemistry, cross-link density, and adhesive–substrate compatibility.

Beyond compositional differences, the manufacturing technique itself might also influence bonding outcomes. The AM materials possess a layer-by-layer structure that tends to show greater surface reactivity following surface treatment, improving adhesive penetration into the superficial matrix [26]. In contrast, SM-produced CAD/CAM blocks undergo extensive industrial polymerization, producing highly converted and uniformly dense surfaces that are less responsive to surface treatment procedures [27]. The findings of the present study are consistent with previous reports comparing the bonding performance of additively and subtractively manufactured restorative materials. Several investigations have similarly demonstrated that 3D-printed definitive resins exhibit superior SBS values compared with CAD/CAM hybrid blocks, largely due to their more responsive resin-rich surface layer and favorable micromechanical interaction following sandblasting [7,21]. In contrast, studies evaluating polymer-infiltrated ceramics and nano-ceramic composites, including Vita Enamic and Cerasmart, have shown that these subtractively produced materials display limited surface alteration after conditioning and consequently lower repair or luting bond strengths [24,28].

Before applying resin composites to repair restorative materials, various surface treatments can play a vital role in achieving micromechanical adhesion [29]. It has generally been reported that mechanical pretreatment results in higher SBS compared to chemical pretreatment [19,30]. Similarly, in the current study, the highest SBS value among surface treatments was obtained with sandblasting. Therefore, the second null hypothesis, that SBS would not change due to surface treatments, was rejected. Among the surface pretreatment protocols, sandblasting significantly improved the bond strength values compared to HF etching and no treatment.

In addition to the main effects of surface treatments, the significant material–surface treatment interaction observed in this study reflects that different restorative materials respond differently to pretreatment approaches. The effect of surface treatment on SBS was material-dependent, particularly among AM restorative materials. For some AM materials (SC and CT), no statistically significant difference was observed between sandblasting and other pretreatments. This trend is consistent with previous studies showing that polymer-rich microstructures could provide basic micromechanical receptivity regardless of the surface treatment [19,31]. HF that selectively dissolves the glassy phase, creating a micro-porous, roughened surface, facilitating resin infiltration and micromechanical interlocking upon adhesive application one of the most used chemical surface treatments [32]. Several studies have shown that HF etching significantly improves the shear or bond strength of ceramic/hybrid materials with untreated surfaces [32,33]. Additively manufactured materials, including SC, VC, and VT, possess a more resin-rich and less densely cross-linked matrix, which facilitates deeper indentation and irregularity formation during abrasion—an effect widely documented in printed restorative polymers [6]. Conversely, etching provided limited benefit, particularly for VE and CS, which is in line with studies reporting that hydrofluoric acid or phosphoric acid produces minimal surface alteration in polymer-infiltrated ceramics and nano-ceramic hybrids due to their compact inorganic networks [28]. The current evidence supports that the efficacy of surface treatment is material-dependent, and while sandblasting is generally advantageous for AM and SM materials, hybrid materials containing a ceramic-dominant phase may respond differently to chemical versus mechanical conditioning. However, it is known that over-etching can lead to excessive dissolution of the matrix, compromising the structural integrity and potentially reducing long-term resistance to fracture [34]. Considering the findings of the current study, HF surface treatment did not cause a significant difference in some specimens compared to the no-surface-treatment group, so adjusting the etch time based on the material may lead to improved bonding.

Several studies have suggested that a minimum shear bond strength of 6–8 MPa is adequate for achieving clinically successful intraoral repair; however, the threshold value of 5 MPa as the minimum requirement for acceptable bonding performance [35,36,37]. In the present study, although there was a significant difference in bond strength between adhesive systems, regardless of the restorative material or surface treatment applied (Etch-and-Rinse: 11.5; Self-Etch: 11.6; Universal: 12.9), successful bonding efficacy was present across all groups. Therefore, the third null hypothesis, that adhesive systems would not affect SBS, was partially rejected. The findings of the present study align with those reported by Çakır et al. [38], indicating that the universal adhesive demonstrated higher SBS compared with both the etch-and-rinse and self-etch systems. The superior performance of the universal adhesive can be attributed to the multifunctional capacity of the 10-MDP monomer. Structurally, 10-MDP contains a hydrophilic phosphate group capable of chemically bonding to metal oxides and zirconia fillers as well as interacting with the glass fillers of other hybrid materials through the formation of stable covalent bonds [39]. Furthermore, the long hydrophobic carbon chain spacer of the 10-MDP monomer contributes to the long-term durability of the interface by reducing water sorption and preventing hydrolytic degradation [39]. Although the presence of 10-MDP, capable of forming chemical bonds with various substrates, is a common feature of universal and self-etch adhesives used in this study, the significant discrepancy in their bond strength values may be attributed to differences in the concentration, purity, or functional efficiency of 10-MDP among the systems, which are not disclosed by manufacturers.

Regardless of the material, all adhesive systems combined with sandblasting exhibited the highest bond strength among the tested surface treatment × adhesive interactions and showed similar values to each other. These findings are consistent with the results of Falcon Aguilar et al. [40] and Yildirim Isik et al. [22] study, which examined the effects of different acid types (phosphoric acid and phytic acid) on bond strength in sandblasted specimens. The similar SBS values obtained across the three adhesive strategies suggest that the micromechanical retention provided by sandblasting may play a more critical role than the surface conditioning achieved with phosphoric acid. Furthermore, among all restorative materials, a significant difference was found in the bond strengths of the adhesive systems used only in VE regardless of the surface treatments. This may be attributed to VE being a polymer-infiltrated ceramic network (PICN).

Single-shade resin composite (ZenChroma) was selected for the repair procedure in this study because single-shade resin composites are specifically formulated to provide reliable adhesion to a wide range of restorative materials, including polymer-infiltrated ceramics, nano-ceramic hybrids, and additively manufactured definitive resins [41]. A recent study demonstrated that the use of single-shade resin composites in repair protocols yields high bond strength values, particularly when combined with appropriate surface treatment [42]. The use of a single-shade resin composite in the present study allowed for the evaluation of material-, surface-treatment-, and adhesive-system-related effects without the influence of resin composites. Similarly, in the present study, all surface-treated specimens exhibited high bond strength.

Various aging methods—such as water immersion, pH cycling, food-simulating liquids, thermal cycling, chewing simulation, and accelerated artificial aging—are commonly used in in vitro dental materials research [43]. Among these, thermal cycling is one of the most widely applied techniques to simulate the thermal stresses encountered in the oral environment [44]. Although thermal cycling is widely used, aging protocols vary considerably across studies. Cycle numbers differ according to the intended simulation period: approximately 500 cycles are often used to represent short-term aging, whereas 10,000 cycles are typically employed to simulate one year of clinical service [44]. In the present study, AM and SM specimens were subjected to 10,000 thermal cycles between 5 °C and 55 °C, with a dwell time of 30 s in each bath and a 15 s transfer time, to approximate one year of intraoral aging. Following aging, various surface treatments and adhesive procedures were applied prior to resin composite repair, and the repaired specimens were analyzed for bond strength and failure modes.

In the present study, the micro shear bond strength test was selected because it enables the evaluation of bonding performance using small, standardized bonding areas, thereby minimizing substrate heterogeneity and stress distribution artifacts commonly encountered in macro-scale shear tests [45]. Failure modes were examined under both light microscopy and SEM to identify the nature of interfacial and substrate-related failures. The use of SEM in particular allows for more precise visualization of resin–substrate interactions, micro-crack propagation patterns, and integrity of conditioned surfaces [46]. This combined evaluation approach is widely recommended in the literature for bonding studies, as it provides a more comprehensive understanding of how microstructural features influence the failure pathway [47]. The microscopic observations of the current study were generally consistent with previously published studies reporting that materials exhibiting higher SBS values tend to demonstrate a greater proportion of cohesive or mixed failures, whereas lower SBS values are frequently associated with adhesive failures at the interface. In the present study, failure mode distribution varied according to material type, surface treatment, and adhesive system. Adhesive failures were more prevalent in control groups or in materials with lower bond strength performance (e.g., CS), reflecting weaker interfacial adhesion. Conversely, sandblasting and etching procedures—particularly when combined with universal adhesives—led to an increase in cohesive–material and mixed failures, indicating improved micromechanical retention and stronger interfacial integrity. This trend aligns with previous research demonstrating that effective surface treatment promotes deeper resin infiltration and stronger chemical interaction, consequently shifting the failure pattern away from purely adhesive separation toward more cohesive modes within the material or resin composite [19].

Limitations include the inherent constraints of an in vitro design, which cannot fully replicate the complex moisture, temperature, and loading cycles of the oral environment. The evaluation was confined to one single-shade resin composite between restorative materials; the inclusion of different structures or viscosity resin composites may yield different outcomes. Additionally, this study did not include alternative surface treatments (silan, bur, and laser, etc.), which could have influenced SBS. Future investigations should incorporate improve understanding of the long-term durability of repairs to digitally fabricated restorations.

## 5. Conclusions

Considering the limitations of the current in vitro study, the findings indicate that sandblasting provides the most reliable improvement in bond strength across all restorative materials and adhesive systems. Surface preparation is critical for CS restorations, as untreated CS consistently exhibits bond strength values below clinically acceptable thresholds, irrespective of the adhesive system used. Given that the highest and comparably high bond strengths were achieved in specimens treated with the universal adhesive across all materials, its use may be considered a reliable clinical choice.

## Figures and Tables

**Table 1:** Composition and manufacturer information of restorative materials and adhesive systems used in the study.

Restorative Materials					
Product	Manufacturer	Composition			LOT
Saremco Cronwtec (SC)	Saremco, Dental AG, Rebstein, Switzerland	Esterification products of 4,4′-isopropylidiphenol, ethoxylated and 2-methylprop-2enoic acid, silanized dental glass, pyrogenic silica, initiators. Total content of inorganic fillers (particle size 0.7 μm) is 30–50 wt%.			E622
VarseoSmile CrownPlus (VC)	BEGO, Bremen, Germany	Esterification products of 4,4′-isopropylidiphenol, ethoxylated and 2-methylprop-2enoic acid, silanized dental glass, methyl benzoylformate, diphenyl(2,4,6-trimethylbenzoyl) phosphine oxide, 30–50 wt%—inorganic fillers (particle size 0.7 μm)			600985
VarseoSmile TriniQ (VT)	BEGO, Bremen, Germany	Bis-GMA, TEGDMA, EBPDMA, Bis-EMA, Silicon dioxide, barium glass, Zirconium oxide, 83.5 wt%, particle size: N/A			601836
Vita Enamic (VE)	Vita Zahnfabrik, Bad Sackingen, Germany	14 wt% methacrylate polymer (UDMA, TEGDMA) and 86 wt% fine-structure feldspathic ceramic network			210450
Cerasmart (CS)	Gc Corp., Tokyo, Japan	71% Barium (300 nm) and silicate glass ceramics (20 nm), Bis-MEPP, UDMA, DMA			2402141
ZenChroma	President Dental GmbH, Allershausen, Germany	UDMA, Bis-GMA, TEMDMA, Glass powder, silicon dioxide, inorganic filler (0.005–3.0 μm)			2024006257
**Adhesive Systems**					
**Product**	**Manufacturer**	**Composition**	**LOT**	**Application Procedure**	
Adper Single Bond 2 (Etch-and-rinse system)	3M ESPE; St. Paul, MN, USA	Bis-GMA; HEMA; Dimethacrylatas; Polyalkanoic acid copolymer; initiators; water; and ethanol	9950798	The composite surface was etched with 37% phosphoric acid (FineEtch 37 Gel; Spident Inc., Incheon, Republic of Korea) for 30 s, rinsed thoroughly, and air-dried. Adper Single Bond was then applied with active rubbing for 20 s, gently air-dried for 5 s, and polymerized for 10 s.	
Clearfil SE Bond (self-etch system)	Kuraray Inc., Kurashiki, Japan	Primer: 10-MDP; HEMA;Hydrophilic Dimethacrylate; Camphorquinone; water. Adhesive: 10-MDP; HEMA; Bis-GMA; Hydrophobic Dimethacrylate; N, N diethanol p-toluidine; Camphorquinone bond; Silanated colloidal silica	Primer: 2G0426 Bond: 2F0861	The primer was applied first, followed by 20 s of gentle air-drying. Then, the bonding agent was applied, air-thinned, and cured for 10 s.	
G-Premio Bond (universal system)	Gc Corp., Tokyo, Japan	10-MDP, MDTP, 4-MET, BHT, acetone, water, dimethacrylate monomer, photoinitiator, silica filler; pH: 1.5	2503037	G-Premio Bond was applied for 20 s, air-thinned for 5 s, and light-cured for 20 s.	

Abbreviations: Bis-GMA: bisphenol A glycerolate dimethacrylate; HEMA: 2-hydroxyethyl methacrylate; UDMA: urethane dimethacrylate; TEGDMA: triethylene glycol dimethacrylate; Bis-EMA: bisphenol A ethoxylate dimethacrylate; 10-MDP: 10-methacryloyloxydecyl dihydrogen phosphate.

## Data Availability

The original contributions presented in the study are included in the article, further inquiries can be directed to the corresponding author.

## Supplementary Materials

The following supporting information can be downloaded at https://www.mdpi.com/article/10.3390/polym18020296/s1: Supplemental Table S1: Comparison of Shear Bond Strength Values According to Material, Surface Treatment, and Adhesive Factors; Supplemental Table S2: Comparison (%) of failure types according to Material, Surface Treatment, and Adhesive Factors.

## Abbreviations

The following abbreviations are used in this manuscript:

SBS	Shear bond strength
AM	Additively manufactured
SM	Subtractively manufactured
SEM	Scanning electron microscope
