# Supplementary material for: Shear Bond Strength of Additively and Subtractively Manufactured CAD/CAM Restorative Materials After Different Surface Treatments and Adhesive Strategies: An In Vitro Study
Source: Polymers (Basel). 2026 Jan 22;18(2):296. doi: 10.3390/polym18020296 (PMC12846001; doi:10.3390/polym18020296)
Supplement: Supplementary file 1 [file polymers-18-00296-s001.zip › polymers-4079853-supplementary.pdf]

Supplemental Table S1. Comparison of Shear Bond Strength Values According to Material, Surface Treatment, and Adhesive Factors

Materials	Surface Treatments	Adhesive Systems			Total
		Etch-and-Rinse	Self-Etch	Universal	
**SC**	**Control**	13.95 ± 0.66^{GHİKLM}	$13.97 \pm 1.09^{AGHİJKLM}$	$12.48 \pm 1.27^{ABCEGHİJKLM}$	13.56 ± 0.56^{CDEF}
	**Etching**	$11.66 \pm 0.68^{ACGHİJKM}$	$13.6 \pm 1.45^{AEGHİJKLM}$	15.25 ± 0.9^{KLM}	13.44 ± 0.58^{CDEF}
	**Sandblastıng**	16.45 ± 0.93^{LM}	$15.06 \pm 1.36^{AGHİJKLM}$	14.59 ± 1.09^{GHİJKLM}	15.35 ± 0.61^{F}
	**Total**	14 ± 0.5^{A}	14.19 ± 0.68^{A}	14.22 ± 0.63^{A}	14.12 ± 0.35^{d}
**VC**	**Control**	7.9 ± 0.94^{ABCDEF}	$8.57 \pm 1.15^{ABCDEFGHİJ}$	$10.7 \pm 0.98^{ABCDEGHİJK}$	9.01 ± 0.59^{AB}
	**Etching**	11 ± 0.81^{ABCEGHİJK}	$12.69 \pm 0.86^{AGHİJKLM}$	$13.74 \pm 1.14^{AGHİJKLM}$	12.27 ± 0.5^{CDE}
	**Sandblastıng**	16.24 ± 0.8^{L}	14.41 ± 0.92^{GHİKLM}	$13.77 \pm 1.13^{AGHİJKLM}$	14.85 ± 0.53^{F}
	**Total**	11.61 ± 0.71^{AB}	11.92 ± 0.67^{AB}	12.69 ± 0.59^{A}	12.08 ± 0.38^{ab}
**VT**	**Control**	$9.49 \pm 1.25^{ABCDEFGHİJK}$	$12.2 \pm 1.33^{ABCEGHİJKLM}$	$13.03 \pm 1.31^{ABCEGHİJKLM}$	11.43 ± 0.71^{BCDE}
	**Etching**	$12.74 \pm 1.21^{ABCEGHİJKLM}$	$11.74 \pm 1^{ABCEGHİJKLM}$	$13.31 \pm 0.95^{AGHİJKLM}$	12.61 ± 0.56^{CDEF}
	**Sandblastıng**	$12.68 \pm 1.14^{AEGHİJKLM}$	13.18 ± 0.63^{GHİJKLM}	15.3 ± 1.12^{HİKLM}	13.65 ± 0.52^{DEF}
	**Total**	11.65 ± 0.72^{A}	12.25 ± 0.5^{A}	13.87 ± 0.59^{A}	12.63 ± 0.36^{b}
**VE**	**Control**	7.05 ± 0.78^{BDEF}	8.48 ± 1.2^{ABCDEFGİJ}	8.68 ± 1.05^{ABCDEFGJ}	7.9 ± 0.55^{A}
	**Etching**	$9.02 \pm 0.98^{ABCDEFGHJ}$	8.74 ± 0.94^{ABCDEFJ}	14.93 ± 0.77^{KLM}	10.89 ± 0.67^{BCD}
	**Sandblastıng**	$11.93 \pm 1.26^{ABCEGHİJKLM}$	16.19 ± 1.5^{GHİJKLM}	14.92 ± 0.97^{İKLM}	14.25 ± 0.68^{EF}
	**Total**	9.21 ± 0.63^{B}	10.81 ± 0.86^{AB}	12.94 ± 0.67^{A}	10.94 ± 0.43^{ac}
**CS**	**Control**	5.28 ± 1.29^{BCDF}	4.85 ± 0.87^{F}	4.68 ± 1.1^{DF}	4.58 ± 0.5^{G}
	**Etching**	$11.52 \pm 1.15^{ABCEGHİJKLM}$	8.73 ± 0.98^{ABCDEFJ}	$12.1 \pm 1.58^{ABCDEFGHİJKLM}$	10.62 ± 0.67^{ABC}
	**Sandblastıng**	16.31 ± 1.17^{KLM}	$13.51 \pm 1.16^{AGHİJKLM}$	$14.03 \pm 1.38^{AEGHİJKLM}$	14.63 ± 0.71^{EF}
	**Total**	10.85 ± 0.99^{AB}	8.84 ± 0.74^{B}	10.12 ± 0.97^{AB}	9.93 ± 0.53^{c}
**Total**	**Control**	8.55 ± 0.56^{A}	9.46 ± 0.59^{AB}	9.88 ± 0.6^{AB}	9.28 ± 0.34^{a}
	**Etching**	11.17 ± 0.39^{B}	10.98 ± 0.49^{B}	13.93 ± 0.44^{C}	12.01 ± 0.27^{b}
	**Sandblastıng**	14.79 ± 0.49^{C}	14.31 ± 0.48^{C}	14.53 ± 0.47^{C}	14.53 ± 0.27^{c}
	**Total**	11.5 ± 0.34^{a}	11.6 ± 0.33^{a}	12.9 ± 0.32^{b}	

	Test statistics	p^x
Material	80.7	**<0.001**
Surface treatment	197.1	**<0.001**
Adhesive	13.2	**0.002**
Material × surface treatment	64.9	**0.001**
Material × adhesive	22.4	**0.007**
Surface treatment × adhesize	16	**0.004**
Material × surface treatment × adhesive	33.9	**0.013**

p^x based on Robust ANOVA; Multiple comparisons were analysed by Bonferroni post-hoc Test. The same lowercase letter (a–c) are not significantly different. The same uppercase letter (A–M) are not significantly different.

Abbreviations: *SC: Saremco Crowntec; VC: VarseoSmile CrownPlus; VT; VarseoSmile TriniQ; VE: Vita Enamic; CS: Cerasmart.*

Supplemental Table S2. Comparison (%) of failure types according to Material, Surface Treatment, and Adhesive Factors

Materials	Surface Treatments	Failure Types	Adhesive Systems			Total	Test statistics	p^x
			Etch-and-Rinse	Self-Etch	Universal			
SC	Control	Adhesive	0 (0)	5 (27.8)	2 (16.7)	7 (15.6)	9.388	0.095
		Cohesive (Material)	10 (66.7)	8 (44.4)	9 (75)	27 (60)		
		Cohesive (Composite)	2 (13.3)	0 (0)	0 (0)	2 (4.4)		
		Mixed	3 (20)	5 (27.8)	1 (8.3)	9 (20)		
	Etching	Adhesive	1 (6.7)	5 (33.3)	1 (6.7)	7 (15.6)	8.341	0.117
		Cohesive (Material)	14 (93.3)	9 (60)	11 (73.3)	34 (75.6)		
		Cohesive (Composite)	0 (0)	0 (0)	1 (6.7)	1 (2.2)		
		Mixed	0 (0)	1 (6.7)	2 (13.3)	3 (6.7)		
	Sandblastıng	Adhesive	0 (0)[a]	0 (0)[a]	5 (33.3)[b]	5 (11.1)	16.262	**0.003**
		Cohesive (Material)	11 (73.3)[a]	11 (73.3)[a]	3 (20)[b]	25 (55.6)		
		Cohesive (Composite)	2 (13.3)	3 (20)	2 (13.3)	7 (15.6)		
		Mixed	2 (13.3)	1 (6.7)	5 (33.3)	8 (17.8)		
	Total	Adhesive	1 (2.2)	10 (20.8)	8 (19)	19 (14.1)	11.358	0.069
		Cohesive (Material)	35 (77.8)	28 (58.3)	23 (54.8)	86 (63.7)		
		Cohesive (Composite)	4 (8.9)	3 (6.3)	3 (7.1)	10 (7.4)		
		Mixed	5 (11.1)	7 (14.6)	8 (19)	20 (14.8)		
VC	Control	Adhesive	12 (80)	10 (66.7)	8 (53.3)	30 (66.7)	3.053	0.589
		Cohesive (Material)	2 (13.3)	4 (26.7)	4 (26.7)	10 (22.2)		
		Cohesive (Composite)	---	---	---	---		
		Mixed	1 (6.7)	1 (6.7)	3 (20)	5 (11.1)		
	Etching	Adhesive	0 (0)[a]	1 (6.7)[a]	7 (46.7)[b]	8 (17.8)	14.973	**0.001**
		Cohesive (Material)	14 (93.3)[a]	12 (80)[ab]	8 (53.3)[b]	34 (75.6)		
		Cohesive (Composite)	1 (6.7)	0 (0)	0 (0)	1 (2.2)		
		Mixed	0 (0)	2 (13.3)	0 (0)	2 (4.4)		
	Sandblastıng	Adhesive	0 (0)[a]	0 (0)[a]	6 (40)[b]	6 (13.3)	17.191	**0.002**
		Cohesive (Material)	10 (66.7)[a]	11 (73.3)[a]	3 (20)[b]	24 (53.3)		
		Cohesive (Composite)	2 (13.3)	2 (13.3)	5 (33.3)	9 (20)		
		Mixed	3 (20)	2 (13.3)	1 (6.7)	6 (13.3)		
	Total	Adhesive	12 (26.7)	11 (24.4)	21 (46.7)	44 (32.6)	9.501	0.136
		Cohesive (Material)	26 (57.8)	27 (60)	15 (33.3)	68 (50.4)		
		Cohesive (Composite)	3 (6.7)	2 (4.4)	5 (11.1)	10 (7.4)		
		Mixed	4 (8.9)	5 (11.1)	4 (8.9)	13 (9.6)		
VT	Control	Adhesive	9 (60)[a]	1 (6.7)[b]	3 (20)[ab]	13 (28.9)	14.916	**0.010**
		Cohesive (Material)	5 (33.3)	6 (40)	9 (60)	20 (44.4)		
		Cohesive (Composite)	0 (0)	3 (20)	0 (0)	3 (6.7)		
		Mixed	1 (6.7)	5 (33.3)	3 (20)	9 (20)		
	Etching	Adhesive	6 (33.3)	3 (20)	6 (40)	15 (31.3)	4.336	0.676
		Cohesive (Material)	9 (50)	8 (53.3)	5 (33.3)	22 (45.8)		

		Cohesive (Composite)	0 (0)	2 (13.3)	1 (6.7)	3 (6.3)		
		Mixed	3 (16.7)	2 (13.3)	3 (20)	8 (16.7)		
	**Sandblastıing**	Adhesive	0 (0)	0 (0)	1 (6.7)	1 (2.2)	3.619	0.847
		Cohesive (Material)	9 (60)	10 (66.7)	7 (46.7)	26 (57.8)		
		Cohesive (Composite)	2 (13.3)	2 (13.3)	4 (26.7)	8 (17.8)		
		Mixed	4 (26.7)	3 (20)	3 (20)	10 (22.2)		
	**Total**	Adhesive	15 (31.3)	4 (8.9)	10 (22.2)	29 (21)	9.536	0.147
		Cohesive (Material)	23 (47.9)	24 (53.3)	21 (46.7)	68 (49.3)		
		Cohesive (Composite)	2 (4.2)	7 (15.6)	5 (11.1)	14 (10.1)		
		Mixed	8 (16.7)	10 (22.2)	9 (20)	27 (19.6)		
**VE**	**Control**	Adhesive	15 (100)^{a}	9 (60)^{b}	11 (73.3)^{ab}	35 (77.8)	8.687	**0.037**
		Cohesive (Material)	0 (0)	4 (26.7)	4 (26.7)	8 (17.8)		
		Cohesive (Composite)	---	---	---	---		
		Mixed	0 (0)	2 (13.3)	0 (0)	2 (4.4)		
	**Etching**	Adhesive	8 (53.3)^{a}	10 (66.7)^{a}	0 (0)^{b}	18 (40)	19.181	**0.001**
		Cohesive (Material)	5 (33.3)^{ab}	3 (20)^{b}	11 (73.3)^{a}	19 (42.2)		
		Cohesive (Composite)	0 (0)	0 (0)	1 (6.7)	1 (2.2)		
		Mixed	2 (13.3)	2 (13.3)	3 (20)	7 (15.6)		
	**Sandblastıing**	Adhesive	5 (33.3)	0 (0)	1 (6.7)	6 (13.3)	10.854	0.069
		Cohesive (Material)	8 (53.3)	9 (60)	7 (46.7)	24 (53.3)		
		Cohesive (Composite)	0 (0)	4 (26.7)	3 (20)	7 (15.6)		
		Mixed	2 (13.3)	2 (13.3)	4 (26.7)	8 (17.8)		
	**Total**	Adhesive	28 (62.2)^{a}	19 (42.2)^{ab}	12 (26.7)^{b}	59 (43.7)	14.247	**0.018**
		Cohesive (Material)	13 (28.9)	16 (35.6)	22 (48.9)	51 (37.8)		
		Cohesive (Composite)	0 (0)	4 (8.9)	4 (8.9)	8 (5.9)		
		Mixed	4 (8.9)	6 (13.3)	7 (15.6)	17 (12.6)		
**CS**	**Control**	Adhesive	15 (100)	15 (100)	15 (100)	45 (100)	---	---
		Cohesive (Material)	---	---	---	---		
		Cohesive (Composite)	---	---	---	---		
		Mixed	---	---	---	---		
	**Etching**	Adhesive	15 (100)	13 (86.7)	11 (73.3)	39 (86.7)	4.705	0.211
		Cohesive (Material)	---	---	---	---		
		Cohesive (Composite)	0 (0)	1 (6.7)	1 (6.7)	2 (4.4)		
		Mixed	0 (0)	1 (6.7)	3 (20)	4 (8.9)		
	**Sandblastıing**	Adhesive	4 (26.7)	0 (0)	4 (26.7)	8 (17.8)	9.581	0.142
		Cohesive (Material)	5 (33.3)	7 (46.7)	2 (13.3)	14 (31.1)		
		Cohesive (Composite)	2 (13.3)	2 (13.3)	5 (33.3)	9 (20)		
		Mixed	4 (26.7)	6 (40)	4 (26.7)	14 (31.1)		
	**Total**	Adhesive	34 (75.6)	28 (62.2)	30 (66.7)	92 (68.1)	6.503	0.361
		Cohesive (Material)	5 (11.1)	7 (15.6)	2 (4.4)	14 (10.4)		
		Cohesive (Composite)	2 (4.4)	3 (6.7)	6 (13.3)	11 (8.1)		

		Mixed	4 (8.9)	7 (15.6)	7 (15.6)	18 (13.3)		
**Total**	**Control**	Adhesive	51 (68)	40 (51.3)	39 (54.2)	130 (57.8)	10.242	0.089
		Cohesive (Material)	17 (22.7)	22 (28.2)	26 (36.1)	65 (28.9)		
		Cohesive (Composite)	2 (2.7)	3 (3.8)	0 (0)	5 (2.2)		
		Mixed	5 (6.7)	13 (16.7)	7 (9.7)	25 (11.1)		
	**Etching**	Adhesive	30 (38.5)	32 (42.7)	25 (33.3)	87 (38.2)	6.239	0.383
		Cohesive (Material)	42 (53.8)	32 (42.7)	35 (46.7)	109 (47.8)		
		Cohesive (Composite)	1 (1.3)	3 (4)	4 (5.3)	8 (3.5)		
		Mixed	5 (6.4)	8 (10.7)	11 (14.7)	24 (10.5)		
	**Sandblastıng**	Adhesive	9 (12)[a]	0 (0)[b]	17 (22.7)[a]	26 (11.6)	35.972	**<0.001**
		Cohesive (Material)	43 (57.3)[a]	48 (64)[a]	22 (29.3)[b]	113 (50.2)		
		Cohesive (Composite)	8 (10.7)	13 (17.3)	19 (25.3)	40 (17.8)		
		Mixed	15 (20)	14 (18.7)	17 (22.7)	46 (20.4)		
